# In Memoriam

**Published:** 1909-12-15

**Authors:** 


### IN MEMORIAM.

Joseph William Wassall, D.D.S., of Chicago, was drowned in Lake Michigan from a sailing yacht September 18th, 1909. Dr. Wassall was a prominent and prosperous dentist of Chicago and a graduate of Mich. College of Dental Surgery of the class of 1881. He was also a graduate of the College of Physicians and Surgeons of Chicago, Class of 1884. For a time Dr. Wassall practiced in St. Petersburg, Russia, and was dentist to the Czar. Most of his life was spent in Chicago where he held a large practice, and was highly esteemed. His death was a great shock to his friends. He leaves two children, a son and a daughter.

